# Adolescent Mice Are Resilient to Alcohol Withdrawal-Induced Anxiety and Changes in Indices of Glutamate Function within the Nucleus Accumbens

**DOI:** 10.3389/fncel.2016.00265

**Published:** 2016-11-18

**Authors:** Kaziya M. Lee, Michal A. Coelho, Hadley A. McGregor, Noah R. Solton, Matan Cohen, Karen K. Szumlinski

**Affiliations:** ^1^Department of Psychological and Brain Sciences, University of California Santa BarbaraSanta Barbara, CA, USA; ^2^Department of Molecular, Cellular and Developmental Biology and the Neuroscience Research Institute, University of California Santa BarbaraSanta Barbara, CA, USA

**Keywords:** binge-drinking, adolescence, group 1 metabotropic glutamate receptors, NMDA receptors, anxiety, depression, alcoholism

## Abstract

Binge-drinking is the most prevalent form of alcohol abuse and while an early life history of binge-drinking is a significant risk factor for subsequent alcoholism and co-morbid affective disorders, relatively little is known regarding the biobehavioral impact of binge-drinking during the sensitive neurodevelopmental period of adolescence. In adult mice, a month-long history of binge-drinking elicits a hyper-glutamatergic state within the nucleus accumbens (Acb), coinciding with hyper-anxiety. Herein, we employed a murine model of binge-drinking to determine whether or not: (1) withdrawal-induced changes in brain and behavior differ between adult and adolescent bingers; and (2) increased behavioral signs of negative affect and changes in Acb expression of glutamate-related proteins would be apparent in adult mice with less chronic binge-drinking experience (14 days, approximating the duration of mouse adolescence). Adult and adolescent male C57BL/6J mice were subjected to a 14-day binge-drinking protocol (5, 10, 20 and 40% alcohol (v/v) for 2 h/day), while age-matched controls received water. At 24 h withdrawal, half of the animals from each group were assayed for negative affect, while tissue was sampled from the shell (AcbSh) and core (AcbC) subregions of the remaining mice for immunoblotting analyses. Adult bingers exhibited hyper-anxiety when tested for defensive marble burying. Additionally, adult bingers showed increased mGlu1, mGlu5, and GluN2b expression in the AcbSh and PKCε and CAMKII in the AcbC. Compared to adults, adolescent mice exhibited higher alcohol intake and blood alcohol concentrations (BACs); however, adolescent bingers did not show increased anxiety in the marble-burying test. Furthermore, adolescent bingers also failed to exhibit the same alcohol-induced changes in mGlu and kinase protein expression seen in the adult bingers. Irrespective of age, bingers exhibited behavioral hyperactivity in the forced swim test (FST) compared to water drinkers, which was paralleled by an increase in AcbC levels of GluN2b. Thus, a 2-week period of binge-drinking is sufficient to produce a hyper-anxious state and related increases in protein indices of Acb glutamate function. In contrast, adolescents were resilient to many of the effects of early alcohol withdrawal and this attenuated sensitivity to the negative consequences of binge drinking may facilitate greater alcohol intake in adolescent drinkers.

## Introduction

Underage binge-drinking is a significant public health concern. Individuals aged 12–20 years old account for 11% of all alcohol consumed in the U.S. (Centers for Disease Control and Prevention, 2014) and over 90% of this alcohol is consumed in the form of binge-drinking episodes. Frequent binge-drinking during the vulnerable developmental period of adolescence can have enduring psychological and neurobiological consequences. Engaging in binge-drinking behavior is a significant risk factor for later development of alcoholism. More specifically, youth who start drinking before age 15 years are five times more likely to develop alcohol dependence later in life than those who begin drinking at or after age 21 years (SAMHSA, 2004).

Adolescence is a dynamic period of cognitive, social and emotional maturation. During this transition from childhood to adulthood, adolescents show distinct behavioral and psychological differences, compared to adults (Dahl, 2004). For instance, adolescents show less positive responses to stimuli of low or moderate incentive value, which is thought to drive adolescents to seek-out stronger reinforcement by engaging in impulsive, risky behaviors such as drug use (Novier et al., 2015). Additionally, adolescents have higher basal levels of anxiety and depression and are more vulnerable to stress (Spear, 2002), which may be a source of negative reinforcement that encourages drug use. Indeed, research has found that perceived stress is one of the strongest predictors of adolescent substance use (Myers and Brown, 1990; Pohorecky, 1991; Wagner, 1993).

Adults and adolescents differ both in their pattern of alcohol consumption and also the consequences of that consumption, especially during the “hangover” period of early withdrawal. Although adolescents typically consumer larger quantities of alcohol per drinking episode than adults, they appear resilient to many of the adverse effects of alcohol that serve as modulatory cues to curb excessive consumption such as locomotor incoordination, subjective intoxication, sedation, and many “hangover” symptoms including, anxiety and dysphoria (Brasser and Spear, 2002; Varlinskaya and Spear, 2004; Spear and Varlinskaya, 2005). In contrast, adolescents appear more sensitive to the positive rewarding effects of alcohol that serve as primary reinforcers of drinking. This combination of permissive/facilitative factors is theorized to drive high alcohol consumption in adolescents (Spear and Varlinskaya, 2005). Although more extensively studied in animal models, these age-related differences and are consistent with reports of greater tolerance and less severe withdrawal symptoms in human adolescent drinkers (Filstead et al., 1989; Martin and Winters, 1998; Winters et al., 1999; Deas et al., 2000).

The nucleus accumbens (Acb) is a basal forebrain structure critically involved in learning, motivation, and reinforcement (Salgado and Kaplitt, 2015). The Acb is composed of the shell and core subregions, which are both anatomically and functionally distinct. The outer shell (AcbSh) subregion is believed to govern the primary positive reinforcing properties of rewarding stimuli (Salgado and Kaplitt, 2015). The AcbSh is part of the extended amygdala, a basal forebrain macrosystem critically involved in emotional processing and regulation (Alheid, 2003), which often undergoes maladaptive plasticity as a result of chronic drug abuse (Koob, 2003). The medial core (AcbC) subregion of the Acb is involved in initiating motivated behavior and mediates the motor “seeking” behaviors associated with a reinforcing stimulus. The AcbC is involved in associative learning and plays a central role in the development and maintenance of operant conditioning. Through its connectivity with the basal ganglia, the AcbC serves as a motor interface in coordinating motivationally salient input with a behavioral output (Corbit et al., 2001).

The Acb is well-characterized with regards to its role in addiction, as virtually all drugs of abuse increase activation of the Acb (Quintero, 2013). Rewarding stimuli, including alcohol and other drugs of abuse, cause an increase in extracellular dopamine and glutamate within the Acb (Szumlinski et al., 2007; Ding et al., 2013), which over time can mold neural circuitry and cause drug-related stimuli to become more salient (Britt et al., 2012). With repeated use, synaptic plasticity within the Acb has been shown to underlie the maintenance and escalation of drug use (Quintero, 2013), as well as craving and the propensity for relapse during withdrawal (Bauer et al., 2013).

In addition to its role in appetitive motivation, the Acb is also involved in aversive motivation (Salamone, 1994). Acb dysfunction has been implicated in a variety of neuropsychiatric disorders characterized by pathologically high negative affect, including bipolar disorder, obsessive-compulsive disorder, anxiety and depression (Shirayama and Chaki, 2006; Salgado and Kaplitt, 2015). In rodents, an increase in Acb glutamate is associated with the manifestation of depressive behaviors (Rada et al., 2003) and reducing Acb activation via NMDA antagonism has anxiolytic effects (Martinez et al., 2002). As such, Acb stimulation is emerging as a promising target for the treatment of both anxiety and depression in the clinical population (Sturm et al., 2003; Bewernick et al., 2010; Nauczyciel et al., 2013). In contrast to the increased excitation of Acb projection neurons typically associated with rewarding stimuli (Kalivas and Nakamura, 1999; Stuber et al., 2011; Britt et al., 2012), it has been shown that glutamatergic excitation of GABAergic interneurons suppresses neurotransmission within the Acb and elicits an aversive state (Qi et al., 2016). Therefore, given the role of the Acb in the regulation of negative affective states, the Acb is also a possible substrate for the aversive properties of drug withdrawal.

The Acb undergoes substantial development during adolescence, as does its major glutamatergic projections from the prefrontal cortex (PFC), which become fully established and strengthened during this critical developmental period (Arain et al., 2013). Insufficient prefrontal control over subcortical activation during adolescence is theorized to underlie a preference for activities requiring low effort but yielding high excitement such as substance use (Kelley et al., 2004). Subcortical hyper-activation also creates a bias towards bottom-up emotional processing, which could contribute to the increased vulnerability to anxiety and depression during adolescence (Casey et al., 2008). Given the immature developmental state of the Acb during adolescence, it is reasonable to speculate that there may also be age-dependent glutamatergic effects of binge-drinking within this region that relates to age-dependent differences in sensitivity to alcohol withdrawal-induced anxiety.

The present study investigated the relation between protein indices of glutamate neurotransmission within the Acb and alcohol withdrawal-induced anxiety. Due to the relatively brief duration of mouse adolescence (Brust et al., 2015), all animals were subjected to a 14-day drinking period. This drinking period is similar to that employed in other studies of adolescent alcohol exposure (Spear, 2000b; Brunell and Spear, 2005; O’Tousa et al., 2013) and enables the extension of prior immunoblotting work (Cozzoli et al., 2012, 2014, 2016) to a shorter history of binge-drinking in adult animals. Approximately 24 h following the final drinking period, animals underwent behavioral testing or tissue collection. We predicted that a history of binge-drinking in adult mice would produce signs of negative affect and increased glutamate-related protein expression in Acb subregions, notably the AcbSh, given its limbic functions. Based on the evidence that adolescents are resilient to many of the aversive properties of alcohol and its withdrawal (Spear and Varlinskaya, 2005), we predicted that symptoms of negative affect would be attenuated or absent in adolescent animals relative to adults. We also hypothesized that this behavioral resilience might be associated with a resistance to binge-induced changes in protein indices of glutamate function within the Acb of adolescent bingers.

## Materials and Methods

### Subjects

This study used two separate cohorts of animals—one for behavioral testing and one for immunoblotting. As a previous study from our laboratory revealed effects of our behavioral testing procedures themselves on cellular activation within Acb subregions (Lee et al., 2015), a separate cohort of animals was used to generate tissue for immunoblotting in order to assess the effects of binge-drinking on protein expression, independent of behavioral-testing confounds. Both cohorts of animals were exposed to identical drinking procedures and each consisted of 48 C57BL/6J (B6) male mice (Jackson Laboratories, Sacramento, CA, USA) that were either 8 weeks (adults; *n* = 24) or 4 weeks (adolescents; *n* = 24) of age at onset of drinking. Within each age group, animals were randomly divided into an alcohol-drinking group (*n* = 12) and a water-drinking group (*n* = 12) and then individually housed in standard, Plexiglas cages, under a 12-h-reverse light/dark cycle (lights off at 10 am), in a temperature-controlled vivarium (23^°^C). Food and water were available *ad libitum*, with the exception of the 2-h alcohol-drinking period, during which time the home cage water bottle was removed. All experiments were conducted in compliance with the National Institutes of Health Guide for Care and Use of Laboratory Animals (NIH Publication No. 80–23, revised 2014) and approved by the IACUC of the University of California, Santa Barbara.

### Drinking-in-the-Dark (DID) Procedures

Animals were subjected to 14 consecutive days of binge-drinking under modified 4-bottle Drinking-in-the-dark (DID) procedures. While earlier studies employed 30-day binge drinking regimens (Cozzoli et al., 2009; Lee et al., 2015), alcohol-access was restricted to 14 days in this study to correspond with the estimated length of adolescence in the mouse (Spear, 2000b). Each day, animals were given simultaneous access to 5, 10, 20 and 40% (v/v) unsweetened ethanol solutions for 2 h beginning at 3 h into the dark phase of the circadian cycle, which corresponds to the time of peak fluid intake. Each day, the amount of alcohol consumed was calculated by bottle weight immediately before and after the drinking period. Control animals received an identical sipper tube of filtered tap water *in lieu* of alcohol. Submandibular blood samples were collected from all alcohol-drinking animals on day 10, immediately following the 2-h drinking period. The timing of the blood collection was selected to ensure that the animals’ intakes had stabilized, while also allowing ample time for recovery prior to behavioral testing. Blood alcohol concentrations (BACs) were determined using an Analox alcohol analyzer (model AM1, Analox Instruments USA, Lunenburg, MA, USA) as per the manufacturer’s instructions.

### Behavioral Testing

A 2-day behavioral test battery commenced approximately 24 h following the final alcohol presentation and consisted of a novel object test and the Porsolt forced swim test (FST) on day 1 and the marble burying test on day 2. These tests were selected based on the results of our prior study demonstrating robust effects of alcohol withdrawal upon the various dependent measures in these paradigms (Lee et al., 2015). All animals completed the novel object test before beginning the FST in order to allow animals to rest between assays. Given the size of our cohorts and the availability of testing equipment, it was not possible to complete all the behavioral testing in a single day. The order of testing was based on considerations regarding the duration of each trial, as well as the instruction from our IACUC which included avoiding additional testing following the FST in order to allow the animals time to fully recover.

#### Novel Object

To test reactivity to a novel object as an index of neophobia- related anxiety (Misslin and Ropartz, 1981; Dulawa et al., 1999), animals were placed in an activity arena measuring 46 cm long × 42 cm wide × 40 cm high. In the center of the arena was placed a novel, inedible, object (we used a patterned ceramic candlestick holder; measuring approximately 6 cm in diameter × 12 cm high). Using AnyMaze^TM^ tracking software (Stoelting Co., Wood Dale, IL, USA), a zone was designated around the novel object and was used to monitor the animals’ interaction with the novel object during the 2-min trial. The number of contacts and total time spent in contact with the novel object, as well as the total distance traveled within the activity arena, were recorded.

#### Porsolt Forced Swim Test

Floating behavior during the Porsolt FST serves as an index of behavioral despair in laboratory animals (Porsolt et al., 1977a) and is a model with high predictive validity for the clinical efficacy of anti-depressant drugs (Porsolt et al., 1977b). Each animal was placed into an 11-cm diameter cylindrical container filled with room-temperature water such that animals were unable to touch the bottom. The latency to first exhibit immobility (defined as no horizontal or vertical displacement of the animal’s center of gravity for 5s+), total time spent immobile, and the numbers of immobile episodes were monitored during a 6-min period using AnyMaze^TM^ tracking software (Stoelting Co., Wood Dale, IL, USA).

#### Marble-Burying

The marble-burying test was used to measure anxiety-induced defensive burying (Njung’e and Handley, 1991). In our paradigm, 12 square glass pieces (2.5 cm^2^ × 1.25 cm tall) were placed in the animals’ home cage, six at each end. Animals were then left undisturbed for 15 min. At the end of the trial, a blind observer recorded the number of marbles at least 75% buried.

### Brain Tissue Collection

**Figure 1:**
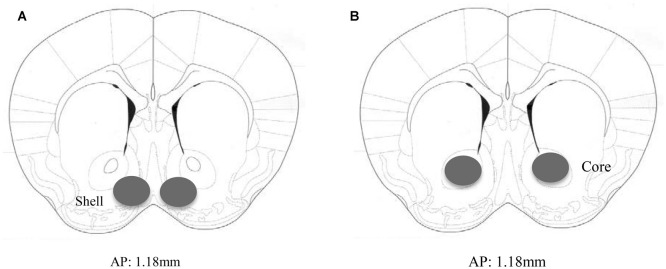
**Immunoblotting sample placement.** Schematic illustrating a coronal section through the accumbens (Acb), highlighting the size and location of the bilateral sampling region used to assay protein changes within the **(A)** AcbSh and **(B)** AcbC.

Animals not subjected to behavioral testing were decapitated approximately 24 h following the final alcohol presentation to mirror the time-frame of that employed in the behavioral study. The brain was cooled on ice and sectioned in 1 mm-thick slices, along the coronal plane, at the level of the striatum using ice-cold razor blades. The AcbSh and AcbC were bilaterally sampled from the slice located approximately 1.18 mm anterior to Bregma, as depicted in the mouse brain atlas of Paxinos and Franklin (2004), using a 18-gauge biopsy needle (as depicted in Figure 1).

### Immunoblotting

Immunoblotting was performed on whole tissue homogenates from the AcbSh and AcbC subregions, following procedures similar to those employed previously by our group (Goulding et al., 2011; Ary et al., 2013; Cozzoli et al., 2014; Lum et al., 2014). Samples were homogenized in a medium containing RIPA buffer (Boston BioProducts, Ashland, MA, USA), Complete Mini-tab Protease Inhibitor Cocktail tablet, sodium fluoride, sodium orthovanadate phosphatase inhibitor cocktail (Sigma-Aldrich, St. Louis, MO, USA). For analysis, 15 μl of homogenized sample was subjected to SDS-polyacrylamide gel electrophoresis using Tris–Acetate gradient gels (3–8%; Invitrogen, Carlsbad, CA, USA) and transferred to polyvinylidene difluoride membranes. Gels were run such that each membrane contained three samples from each age/drinking group. Membranes were preblocked with tris-buffered saline containing 0.1% (v/v) Tween 20 and 5% (w/v) nonfat dried milk powder for 1 h before overnight incubation with the following rabbit primary antibodies: mGlu1 (Synaptic Systems, Göttingen, Germany; 1:1000 dilution), mGlu5 (Millipore, Temecula, CA, USA; 1:1000 dilution), GluN2A and GluN2B (both from Calbiochem, San Diego, CA, USA; 1:1000 dilution), CAMKII (Millipore, Temecula, CA, USA; 1:1000 dilution) and Thr286 phosphorylated CAMKII (pCAMKII; Cell Signaling Technology, Beverly, MA, USA; 1:1000 dilution), PKCε (Santa Cruz Biotechnology, Dallas, TX; 1:500 dilution) and Ser729 phosphorylated PKCε (pPKCε; Santa Cruz Biotechnology, Dallas, TX; 1:500 dilution), and calnexin (Enzo Life Sciences, Farmingdale, NY; 1:1000 dilution) for standardization.

Membranes were washed, incubated with a horseradish peroxidase-conjugated goat anti-rabbit secondary anti-body (Jackson ImmunoResearch Laboratories, West Grove, PA; 1:100,000 dilution) for 90 min, washed again, and immunoreactive bands were detected by enhanced chemiluminescence (ECL Plus; Amersham Biosciences, Inc., Piscataway, NJ). Levels of immunoreactivity were quantified by integrated density using Image J (NIH, Bethesda, MD) and standardized to each animal’s respective calnexin signal. These values were then averaged across the adult water control samples within each gel (*n* = 3/gel) and all bands on that gel were normalized as percent of the average control value. To obtain an index of kinase activation, the density × area measurements for each phospho-protein was also normalized to that of its corresponding non-phosphorylated protein prior to expressing the data as a percent average of the controls on each gel.

Our proteins of interest were selected based on previous work from our lab demonstrating that binge-drinking history upregulates these protein indices of excitatory neurotransmission including mGlu1/5, NR2A/B, and the downstream effector protein kinase C epsilon (PKCε; Szumlinski et al., 2008; Cozzoli et al., 2009, 2016; Goulding et al., 2011), which are believed to promote a “pro-alcoholic” phenotype (Szumlinski et al., 2008; Kalivas et al., 2009; Cozzoli et al., 2012). For the present study, we also included plasticity-related calcium/calmodulin-dependent protein kinase II (CAMKII) in our analysis due to its recent implication in the maintenance of alcohol consumption, as well as negative affective states (Easton et al., 2013a; Zhao et al., 2015). Prior work from our group indicates that alcohol-drinking history increases glutamate-related protein expression selectively within the AcbS (Szumlinski et al., 2008; Cozzoli et al., 2009, 2012, 2016; Goulding et al., 2011). Thus, we measured protein expression within both the Acb shell and core in the present study with the expectation that the AcbS would show a greater number and larger changes in protein levels, relative to the adjacent, but functionally distinct, AcbC.

### Statistical Analysis

Statistical analyses of all behavioral and immunoblotting data were conducted using between-subjects two-way analysis of variance (ANOVAs), along with planned comparisons to assess group differences based on treatment and age using Fisher’s LSD tests for simple main effects. α= 0.05 for all analyses. Statistical outliers were identified using the ±1.5 × IQR rule and excluded from analyses. No more than two outliers were present per group, resulting in n’s of 10–12 per age/drinking group. All calculations were performed using SPSS v.21 statistical software (IBM, 2012).

## Results

### Alcohol Consumption

**Figure 2:**
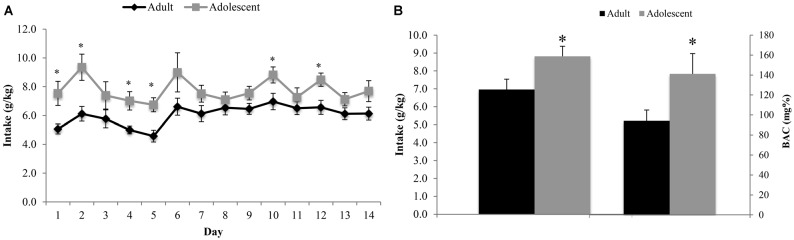
**Adolescents consume more alcohol than adults. (A)** Over the 14-day drinking period, adolescent animals consumed significantly more alcohol than adults (*p* < 0.05). The average intake across all 14 days was 6.13 ± 0.19 g/kg for adults and 7.68 ± 0.23 g/kg for adolescents. **(B)** Blood samples were collected on day 10 of drinking, immediately following the 2 h drinking period. Adolescent animals consumed more alcohol on this day and had a higher average blood alcohol concentrations (BACs) than their adult counterparts. The data represent the means ± SEMs of 10–12 mice/group, excluding statistical outliers. *p < 0.05 vs. adults.

The ANOVA revealed a significant between-subjects effect of age (*F*_(1,22)_ = 7.54, *p* = 0.012), with adolescent animals consuming significantly more alcohol than adults across the entire 14-day drinking period (Figure 2A). The analysis of the blood samples collected immediately following the 2-h drinking period on day 10 yielded an average BAC of 94.12 ± 10.83 mg/dl in adult mice exhibiting an average alcohol intake of 6.97 ± 0.57 g/kg and an average BAC of 141.17 ± 10.23 mg/dl in adolescents exhibiting an average alcohol intake of 8.82 ± 0.56 g/kg. Based on the NIAAA criteria of >80 mg/dl BAC (National Institute on Alcohol Abuse and Alcoholism, 2004), both age groups were engaged in binge drinking. However, adolescent mice binge-drank more alcohol (*t*_(22)_ = 2.32, *p* = 0.029) with higher resulting BACs (*t*_(22)_ = 2.13, *p* = 0.043; Figure 2B) compared to their adult counterparts on day 10. There was no significant difference in alcohol intake or body weight between the animals tested for behavior vs. those used for immunoblotting.

### Behavioral Testing

#### Novel Object Test

**Figure 3:**
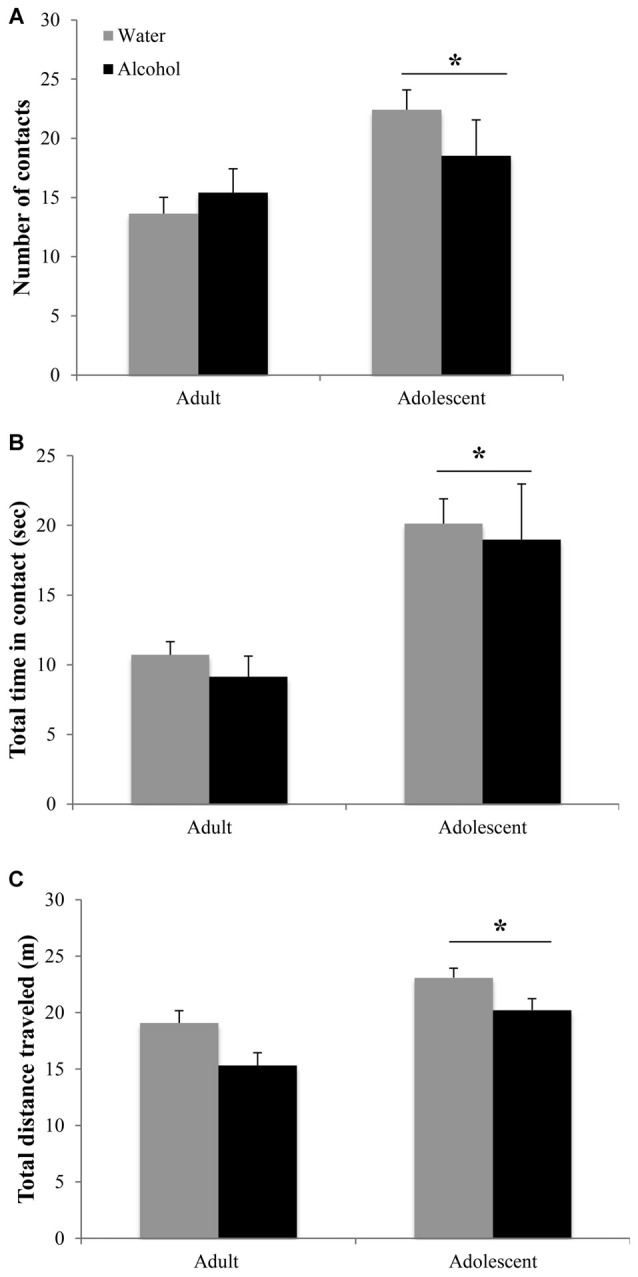
**Differences in the novel object test are age-dependent but alcohol-insensitive. (A)** Adolescent mice made more contacts with the novel object and **(B)** spent more total time in contact with the object during the 2 min trial. **(C)** Adolescent animals also showed general locomotor hyperactivity compared to adults. The data represent the means ± SEMs of 10–12 mice/group. *p < 0.05 vs. adults.

Adolescent animals were more interactive and hyperactive in the novel object test, compared to adults (Figure 3). Adolescents made more object contacts (age effect: *F*_(1,44)_ = 7.47, *p* = 0.009; Figure 3A) and spent more time in contact with the novel object during the 2-min trial (age effect: *F*_(1,43)_ = 14.46, *p* < 0.001; Figure 3B), irrespective of their prior binge-drinking history (no Treatment effects or interactions for either variable, *p*’s > 0.05). Adolescents also traveled a greater overall distance in the enclosure (age effect: *F*_(1,42)_ = 19.36, *p* < 0.001; Figure 3C), although alcohol-drinking animals of both ages were hypoactive compared to water-drinking controls (Treatment effect: *F*_(1,42)_ = 10.74, *p* = 0.002; interaction: *p* > 0.05).

#### Marble Burying Test

**Figure 4:**
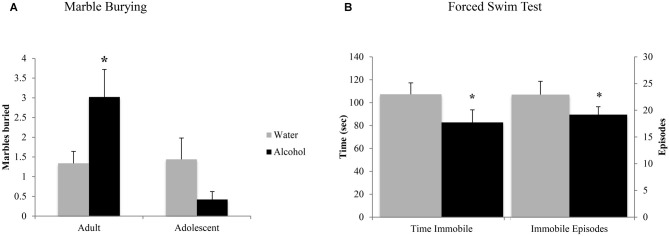
**Withdrawal from binge drinking produces mixed effects on behavioral measures of negative affect. (A)** In the marble burying test, there was an age × treatment interaction showing that adult drinkers buried more marbles compared to their water-drinking counterparts while adolescents trended toward burying less. **(B)** In the forced swim test (FST), an age-independent main treatment effect showed that alcohol-drinking animals showed significantly reduced immobility compared to water drinkers, both in number of immobile episodes and total time spent immobile. The data represent the means ± SEMs of 10–12 mice/group. *p < 0.05 vs. respective water control.

In the marble burying test, an age × treatment interaction (*F*_(1,43)_ = 4.10, *p* = 0.049; Figure 4A) was detected. Deconstruction of the interaction revealed that adult alcohol drinkers buried more marbles than age-matched water controls (LSD *p* = 0.042), while adolescent alcohol drinkers trended toward burying less marbles (LSD *p* = 0.09).

#### Forced Swim Test

As both adult and adolescent alcohol-drinkers showed comparable reductions in immobility, compared to their respective controls, the data from this test were collapsed across age to highlight the alcohol-water difference (Figure 4B). Alcohol-drinkers spent less time immobile (Treatment effect: *F*_(1,43)_ = 4.31, *p* = 0.043) and had fewer immobile episodes (Treatment effect: *F*_(1,42)_ = 4.33, *p* = 0.044), compared to water-drinkers. There were no age-related differences in behavior or interactions between age and prior drinking history (all *p*’s > 0.05).

### Western Blotting

#### Accumbens Shell

**Figure 5:**
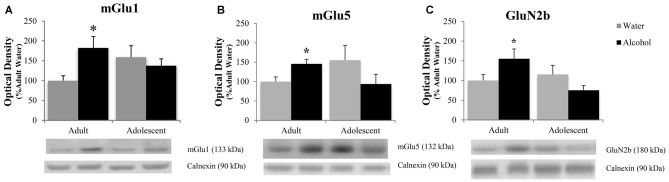
**Adult animals are more vulnerable than adolescents to binge-induced increases in protein expression within the AcbSh.** Adult drinkers showed a significant increase in **(A)** mGlu1, **(B)** mGlu5 and **(C)** GluN2B at 24 h withdrawal that was not present in adolescent drinkers. The data in panels** (A–C)** represent mean ± SEM of 10–12 mice/group; *p < 0.05 vs. respective water control.

The positive experimental outcomes from the immunoblotting study of the AcbSh are presented in Figure 5. Significant age × treatment interactions were detected for mGlu1 (*F*_(1,40)_ = 5.17; *p* = 0.028; Figure 5A), mGlu5 (*F*_(1,41)_ = 6.58; *p* = 0.014; Figure 5B), and GluN2B (*F*_(1,41)_ = 5.11; *p* = 0.029; Figure 5C). LSD analysis of simple main effects revealed that water-drinking adolescents had higher basal mGlu1 (*p* = 0.03), and mGlu5 expression (*p* = 0.04) compared to water-drinking adults. Additionally, adult bingers exhibited a significant alcohol-induced increase in mGlu1 (*p* = 0.033), mGlu5 (*p* = 0.042), and GluN2b (*p* = 0.037) at 24 h withdrawal. In contrast, adolescent mice showed no alcohol-induced change in mGlu1, mGlu5, or GluN2b (all *p*’s > 0.05). Non-significant immunoblotting results are summarized in Table 1.

**Table 1:** **Summary of non-significant immunoblotting results from the AcbSh and AcbC**.

	Adults		Adolescents	
	Water	Alcohol	Water	Alcohol
**AcbSh**
GluN2A	100.0 ± 18.8	#170.48 ± 29.1	154.9 ± 35.6	141.3 ± 25.1
PKCε	100.0 ± 10.1	127.67 ± 23.7	118.5 ± 18.6	80.9 ± 6.2
pPKCε	100.0 ± 11.0	99.7 ± 14.7	86.2 ± 11.8	67.4 ± 9.4
CAMKII	100.0 ± 9.5	97.2 ± 17.0	115.7 ± 14.4	116.3 ± 26.6
pCAMKII	100.0 ± 5.6	117.2 ± 25.9	105.7 ± 16.6	128.9 ± 15.9
**AcbC**
mGlu1	100.0 ± 16.4	85.3 ± 13.1	88.0 ± 7.3	90.2 ± 6.3
mGlu5	100.0 ± 4.9	108.2 ± 24.9	127.3 ± 19.3	107.0 ± 17.1
GluN2A	100.0 ± 7.3	138.4 ± 24.5	120.9 ± 12.5	118.7 ± 24.9
PKCε	100.0 ± 12.6	99.9 ± 10.0	115.3 ± 12.0	107.0 ± 16.4
pCAMKII	100.0 ± 18.8	#161.3 ± 28.1	100.9 ± 13.5	97.0 ± 6.6

*(A) In the AcbSh, there was a trend toward an alcohol-induced increase in GluN2A in adult bingers and a decrease in PKCε in adolescent bingers. (B) In the AcbC, there was a trend toward an alcohol-induced increase in pCAMKII in adult bingers. The data represent mean ± SEM of 10–12 mice; #p < 0.10 vs. respective water control*.

#### Accumbens Core

**Figure 6:**
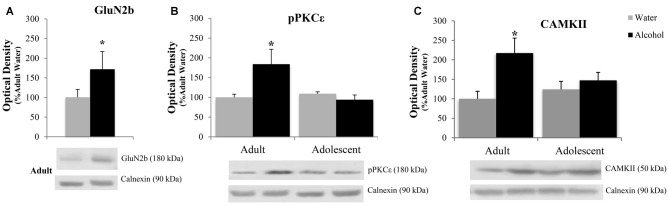
**Adult animals are more vulnerable than adolescents to most binge-induced increases in protein expression within the AcbC. (A)** A main treatment effect of alcohol showed a binge-induced increase in GluN2b independent of age. **(B)** Adult drinkers showed a significant increase in phosphorylated PKCe (pPKCe) at 24 h withdrawal that was not present in adolescent drinkers, which resulted in a similar increase in the phospho: total PKCe ratio (data not shown). **(C)** The analysis of variance (ANOVAs) showed a main treatment effect of alcohol on CAMKII expression. However, further analysis revealed that this effect was primarily due to the significant increase in alcohol-drinking adults. The data in panels **(A–C)** represent mean ± SEM of 10–12 mice/group; *p < 0.05 vs. respective water control.

The positive experimental outcomes from the immunoblotting study of the AcbC are presented in Figure 6. In the AcbC, alcohol-drinking animals showed an age-independent increase in GluN2B (Treatment effect: *F*_(1,430)_ = 4.61; *p* = 0.038; no Age effect or interaction, *p*’s > 0.05; Figure 6A). We also observed an age × treatment interaction for total pPKCε (*F*_(1,40)_ = 4.22; *p* = 0.047; Figure 6B), which mirrored the results of the activated:total PKCε ratio (*F*_(1,43)_ = 4.12; *p* = 0.049) reflecting an alcohol-induced increase in pPKCε in adult drinkers (LSD *p* = 0.049) that was not present in adolescent drinkers (LSD *p* > 0.05). The 2-way ANOVA also revealed an alcohol-dependent increase in CAMKII (treatment effect: *F*_(1,41)_ = 7.44; *p* = 0.009; Figure 6C) and although, the age × treatment interaction was shy of statistical significance (*p* = 0.07), inspection of the results argue that the treatment effect is being driven primarily by the data from the alcohol-drinking adults. Indeed, LSD planned comparisons showed a significant increase in adult drinkers compared to water controls (*t*_(21)_ = 2.80; *p* = 0.011), but no significant difference between alcohol- and water-drinking adolescents (*p* > 0.05).

## Discussion

Cessation of excessive alcohol consumption often results in a dysphoric state to which adolescent drinkers appear less susceptible (Spear and Varlinskaya, 2005). This age-dependent insensitivity to the affective consequences of early alcohol withdrawal is apparent in both humans and animal models of alcoholism (Doremus et al., 2003; Spear and Varlinskaya, 2005). The underlying neurobiological mechanisms contributing to adolescent resilience to withdrawal-induced dysphoria is not well understood, particularly within the context of the most prevalent pattern of alcohol drinking exhibited by adolescents—binge drinking. As such, the present study employed behavioral and immunoblotting procedures to determine the interactions between the subject factors of age and binge-drinking history with respect to emotionality and indices of glutamate transmission within Acb subregions. While correlational in nature, the study outcomes provide novel evidence that the age of binge-drinking onset is an important subject factor that contributes to both alcohol withdrawal-induced negative affect and changes in Acb glutamate, the causal relation between which will be a major focus of future studies.

Consistent with prior literature characterizing adolescent drinking behavior in both humans (National Institute on Alcohol Abuse and Alcoholism, 2002) and laboratory rats (Doremus et al., 2005; Spear and Varlinskaya, 2005; Vetter et al., 2007), adolescent mice in our study consumed significantly more alcohol than adults across the 14-day drinking period, with higher resulting BACs following the 2-h drinking session. Notably, both adult and adolescent animals attained intoxicating “binge” levels of drinking, however that of the adolescents was significantly greater (National Institute on Alcohol Abuse and Alcoholism, 2015).

### Behavioral Dysregulation at 24 h Withdrawal from Binge-Drinking

When assayed at 24 h withdrawal, the adult bingers in this study exhibited hyper-anxiety in the marble burying test, which is consistent with our previous data showing an increase in marble burying in adult mice with a 30-day history of binge drinking (Lee et al., 2015). This suggests that even a relatively brief, 2-week period, of binge-drinking is sufficient to elicit a negative affective state in adult animals. In contrast, despite consuming more alcohol and achieving higher BACs, adolescent alcohol-drinkers showed resilience to withdrawal-induced anxiety in this paradigm, as indicated by no alcohol-induced increase in marble burying. These results corroborate those of other studies showing resilience to the anxiogenic effects of acute alcohol withdrawal in adolescent rats when assayed by the elevated plus maze and social interaction test (Doremus et al., 2003; Varlinskaya and Spear, 2004). Thus, the results of the present study complement existing work in the field by extending these findings to a mouse model of voluntary binge-drinking and demonstrating the sensitivity of an additional behavioral assay to the anxiogenic effects of alcohol withdrawal.

The adolescent mice in our study were overall more interactive and hyperactive in the novel object test compared to adult mice. Elevated novelty-preference and novelty-induced locomotor hyperactivity in adolescents vs. adults is associated with greater impulsivity and a predisposition toward sensation-seeking (Stansfield and Kirstein, 2006). In humans, these traits are strongly predictive of engaging in risk-taking behaviors and substance abuse in human adolescents (Spear, 2000a, 2010; MacPherson et al., 2010). Given that locomotor suppression is a common symptom of acute alcohol withdrawal (Kliethermes et al., 2004; Kliethermes, 2005), it is not surprising that both adult and adolescent bingers exhibited less locomotor activity in the novel object test, compared to their respective age-matched water controls. Although, overall, adolescents showed greater locomotor hyperactivity, compared to adults, both age groups were susceptible to this alcohol withdrawal-induced hypo-activity. This outcome contrasts with that from our prior study in which adult mice binge-drank alcohol for 30 days and indicates that, unlike marble burying, the novel object test does not appear sensitive to changes in anxiety induced by a 14-day history of binge-drinking and it remains to be determined whether the subject factors of age and binge-drinking interact in this paradigm in animals with more prolonged history of excessive alcohol intake.

However, consistent with our previous study in adults (Lee et al., 2015), all binging animals, irrespective of age, exhibited hyper-activity in the FST, as evidenced by reduced time spent immobile and a lower number of immobile episodes, relative to water controls. Although the FST is typically used as an assay of depression, based on increased floating behavior (Porsolt et al., 2001), we have reliably observed a decrease in floating behavior in animals with a history of binge-drinking. We have previously interpreted this hyperactive swimming as reflecting panic in alcohol-withdrawn mice, given that panic is often characterized in laboratory animals using measures of motivated escape in which the animal is actively engaged in fleeing from aversion or a perceived proximal threat (Sena et al., 2003; Campos et al., 2013). In humans, panic is considered an anxiety-related condition with a distinct presentation and symptom profile (Craske et al., 2010; American Psychiatric Association, 2013). Panic disorder shares a high comorbidity with alcohol use disorders (Cowley, 1992; Marshall, 1997) and furthermore, frequent bouts of intoxication and withdrawal are capable of eliciting neuroadaptations that may precipitate symptoms of panic (Cosci et al., 2007).

Despite the somewhat unconventional interpretation of our FST data, the fact that these differences can be observed as early as 24 h following a 2-week history of binge-drinking argues that this assay is particularly sensitive to this pattern of excessive alcohol consumption. However, as both binge-drinking adolescents and adults exhibited a similar behavioral profile in this assay, withdrawal-induced hyperactivity in the FST does not appear to be sensitive to differences in the age of binge-drinking onset. It is also plausible that the failure to observe an age-dependent effect of binge-drinking history in this test reflects the severity of the stressor, which is both psychological and physiological in nature and potentially life-threatening (vs. encountering novel, but benign, objects such as marbles). These data indicate that binging adolescents are not wholly impervious to alcohol withdrawal-induced anxiety of a panic-like nature and that factors associated with the nature of stressor may be critical in determining whether or not binging adolescents exhibit withdrawal-induced behavioral dysregulation.

### Changes in Protein Expression within the Acb During Withdrawal from Binge-Drinking

To complement our behavioral data and expand upon our prior work (Lee et al., 2015), we examined changes in protein expression within the Acb, a structure known to be sensitive to drug-induced neuroadaptations. We know that alcohol-induced dysregulation of excitatory signaling within the Acb is highly implicated in the maintenance and escalation of alcohol consumption, including binge-drinking (Szumlinski et al., 2005, 2007, 2008; Cozzoli et al., 2009, 2012, 2016; Lum et al., 2014); however, virtually no studies have assessed the role of the Acb in withdrawal-induced negative affect despite this structure’s involvement in emotional circuitry.

#### Glutamate Receptor Expression in the AcbSh Parallels Withdrawal-Induced Anxiety

Similar to our previous studies in which mice were subjected to months-long drinking procedures (Szumlinski et al., 2008; Cozzoli et al., 2012, 2016), we found that binge-alcohol experience significantly increased mGlu1/5 and GluN2b within the AcbSh. However, we failed to replicate previous work showing an increase in AcbSh PKCε priming (Cozzoli et al., 2016). This discrepancy are likely attributable to differences in the duration of binge-exposure (14 vs. 30 days) between the two studies, suggesting that alcohol-induced protein changes are, not surprisingly, experience-dependent and manifest differentially over the course of brief to prolonged exposure. The increased expression of mGlu1, mGlu5, and GluN2b expression in the AcbSh of adult bingers paralleled the hyper-anxious behaviors displayed by adult bingers in the marble burying test and FST. Given the well-established role of glutamate in anxiety (Bergink et al., 2004; Swanson et al., 2005; Simon and Gorman, 2006; Kotlinska and Bochenski, 2008; Koltunowska et al., 2013), these results were consistent with our hypothesis that withdrawal-induced anxiety would be associated with increased protein indices of glutamatergic transmission within the AcbSh, thereby further implicating AcbSh excitability in withdrawal-induced negative affect. The AcbSh also receives significant glutamatergic innervation from the amygdala, which is highly susceptible to alcohol-induced perturbation and is known to mediate many aspects of withdrawal-induced negative affect (Christian et al., 2012; Gilpin et al., 2015). Therefore, increased glutamate-related protein expression could render the AcbSh hypersensitive to excitatory innervation from the amygdala and perpetuate alcohol-induced dysfunction within the emotional circuitry of the extended amygdala.

Consistent with the literature (reviewed in Crews et al., 2007), the adolescent water-drinking controls exhibited higher basal glutamate receptor expression in the AcbSh, compared to control adults. These receptors have been shown to be important in all aspects of alcohol consumption including drug-seeking, maintenance and escalation of intake, and relapse (reviewed in Tsai et al., 1995; Gonzales and Jaworski, 1997; Kalivas et al., 2009). Our lab has previously shown higher basal mGlu1 expression in the AcbSh of two distinct lines of mice selectively bred to binge-drink high amounts of alcohol (Cozzoli et al., 2009, 2012). Therefore, it is likely that hypersensitivity of these “pro-binge” receptors is an underlying factor contributing to the greater alcohol consumption seen in adolescent vs. adult mice. Interestingly, the binge-induced increases in receptor expression seen in adult animals were not present in the adolescent bingers. These results paralleled the behavioral data from the marble burying test and provide additional evidence in support of adolescent resilience to binge-induced behavioral and neurobiological abnormalities.

#### Kinase Expression in the AcbC Parallels Withdrawal-Induced Anxiety

Adult bingers showed an increase in CAMKII and activated PKCε in the AcbC at 24 h withdrawal, which also tracked with the behavioral data from the marble burying test. Similar to the results from the AcbSh, these protein changes were not present in adolescent bingers. Although there were no changes seen in mGlu1/5 receptor expression, both PKCε and CAMKII are downstream substrates of group1 mGlu activation and have both been implicated in alcohol-induced neural adaptations (Lee and Messing, 2008). PKCε is an emergent target of interest in the treatment of alcoholism given its role in the maintenance and escalation of drinking (Gass and Olive, 2009; Lesscher et al., 2009; Cozzoli et al., 2016) and its ability to influence hypnotic sensitivity to alcohol (Choi et al., 2002). PKCε is also of interest in the treatment of anxiety due to its ability to regulate GABA receptor function (Gordon, 2002). Additionally, animal studies that show PKCε knockout mice are less anxious than wild types (Hodge et al., 2002). Therefore, it is plausible that an alcohol-induced increase in activated PKCε may contribute to a hyperanxious state during withdrawal.

CAMKII is a critically important regulator of glutamatergic signaling. CAMKII interacts directly with both metabotropic and ionotropic glutamate receptors and plays an essential role in controlling receptor function, trafficking, and localization (Mao et al., 2014). As such, CAMKII is a protein marker of synaptic plasticity and is essential for long-term potentiation. CAMKII-dependent modulation of AMPA receptor trafficking within the Acb is theorized to contribute to the maladaptive plasticity resulting from other drugs of abuse (Pierce and Wolf, 2013; Scheyer et al., 2016). Studies have shown that CAMKII plays a significant role in addiction as a mediator between accumbal DA and glutamate (Anderson et al., 2008). Accordingly, CAMKII is associated with craving and relapse for a variety of drugs including morphine (Liu et al., 2012), cocaine (Easton et al., 2014), and alcohol (Zhao et al., 2015). Alcohol has been shown to increase CAMKII-dependent phosphorylation of AMPA receptors within the Acb (Cannady et al., 2016) and elevated Acb CAMKII is theorized to contribute to the reinforcing properties of alcohol (Easton et al., 2013a,b). Although the involvement of Acb CAMKII in emotional processes has not been well-defined, CAMKII is thought to play a role in anxiety through its enhancement of AMPA and NMDA activity, as both AMPA and NMDA blockade within the Acb has anxiolytic effects (Martinez et al., 2002).

In addition to these kinase changes, we also found an age-independent increase in GluN2b in the AcbC, which resembled the behavioral changes seen in the FST. Given the role of the AcbC as a “limbic-motor interface” integrating motivation and action (Mogenson et al., 1980), the increased glutamate receptor expression in the AcbC could render animals hypersensitive to stressful environmental conditions and primed to flee from aversive or threatening situations in a panic-like state. In support of this interpretation, a study characterizing the behavior of mGlu2 knockout mice showed that these animals displayed hyperlocomotion under the stressful conditions of the FST, which coincided with enhanced glutamate signaling in the Acb (Morishima et al., 2005). However, it is also possible that these AcbC protein changes reflect adaptations related to conditioned aspects of drug reinforcement, many of which are mediated by the AcbC. For example, AcbC NMDA receptors are necessary for alcohol conditioned place-preference (Gremel and Cunningham, 2009) and AcbC NMDA signaling has been shown to mediate aversion-resistant alcohol consumption (Seif et al., 2013). Additionally, increased glutamatergic transmission within the AcbC is associated with cue-induced reinstatement of alcohol seeking (Gass et al., 2011).

Individual housing conditions are capable of eliciting behavioral and/or neurochemical changes (Brain, 1975; Goldsmith et al., 1978; Hilakivi et al., 1989), particularly in adolescent animals (Robbins et al., 1996; Weintraub et al., 2010; Amiri et al., 2015). Although individual housing could be a potential confounding factor for our results, other researchers have demonstrated that elevated alcohol consumption and resilience to withdrawal-induced anxiety in adolescent animals is not a function of isolation stress/individual housing (Brunell and Spear, 2005). Additionally, our lab has completed subsequent unpublished replicates of this study design in which animals were group housed and only separated during the drinking period. These experiments have yielded comparable alcohol intake and behavioral data. Although tissue samples were also collected from these animals, the immunoblotting has not yet been processed. Therefore, it will be interesting to see what, if any, effect single-housing stress has on binge-induced changes in protein expression.

Although our binge-induced changes in kinase-related proteins did not perfectly align with receptor changes in either the AcbSh or Acb, changes in total protein expression do not always correspond to changes in receptor function, nor do they necessarily indicate the behavioral relevance of the receptor for alcohol intake. Indeed, our laboratory has detected alcohol-induced changes in Acb levels of mGlu5 in some (Figure 5B; Goulding et al., 2011; Cozzoli et al., 2012), but not all studies (Szumlinski et al., 2007), which may reflect differences in the subregions examined, route of administration/drinking paradigm employed. Nevertheless, intact mGlu5 function within Acb subregions, notably the AcbSh, is important for binge-drinking behavior (Cozzoli et al., 2009; Besheer et al., 2010; Sinclair et al., 2012). Therefore, behavioral differences could be driven by changes in receptor function that are not reflected in changes in total protein expression. Given the present observation of an age-related difference in AcbSh mGlu5 expression, it is important for follow-up studies to causally relate to the manifestation of hyper-anxiety during early alcohol withdrawal to mGlu5 function within Acb subregions and determine age-related differences therein.

It is noteworthy that the AcbSh and AcbC show distinct profiles of binge-induced protein changes. These regions, while highly interconnected, are both functionally and anatomically distinct and serve unique roles in the neurobiology of drug abuse (Di Chiara, 2002; Quintero, 2013; Salgado and Kaplitt, 2015). Given that the Acb does not respond in unison to binge-induced dysregulation, exploring the functional significance and of these region-specific consequences of binge drinking are worthy of future investigation. It is also important to acknowledge that these neurobiological changes found in the Acb may not be functionally related to our behavioral data and perhaps a different brain region such as the amygdala or BNST is the primary mediator of these changes. This study provides a necessary initial characterization of distinct, age-dependent differences in alcohol-induced neuroadaptations and withdrawal phenotype in adult and adolescent male mice following a relatively brief history of binge drinking. However, further research is necessary to establish a causal relationship between alcohol-induced changes in the Acb and withdrawal-induced negative affect.

### Conclusion

In this study, we demonstrate that a 2-week voluntary binge-drinking experience is sufficient to increase behavioral signs of anxiety in adult male mice, concomitant with increased indices of excitatory neurotransmission within Acb subregions. Despite exhibiting higher basal glutamate receptor expression and greater alcohol intake than adult mice, adolescents appear more resilient than adults to particular affective and neurobiological consequences of binge drinking during early withdrawal. Given that glutamatergic synapses in the Acb are not yet fully developed in adolescence, this immaturity may be protective and render them less susceptible to alcohol-induced perturbation compared to adult animals, as was demonstrated in the present study. However, engaging in binge drinking during adolescence could adversely affect the maturation of this system and shape developing neural circuitry in such a way that creates a predisposition to addiction later in life. Additionally, this study presents an intriguing possibility for the involvement of excitatory signaling within the Acb in withdrawal-induced anxiety in adult bingers, which warrants further investigation. Thus, ontogenetic differences exist in vulnerability to alcohol-induced neuroplasticity within Acb that could contribute to age-related differences in binge drinking behavior and future addiction vulnerability.

## Author Contributions

KML and KKS: designed the experiments. KML, MAC, HAM, NRS and MC: conducted the experiments. KML: composed the manuscript. KML and KKS: edited the manuscript.

## Funding

Funding provided by National Institute on Alcohol Abuse and Alcoholism (NIAAA) grants AA016650 and AA024044 to KKS.

## Conflict of Interest Statement

The authors declare that the research was conducted in the absence of any commercial or financial relationships that could be construed as a potential conflict of interest.
